# Adsorption and Release of Growth Factors from Four Different Porcine-Derived Collagen Matrices

**DOI:** 10.3390/ma13112635

**Published:** 2020-06-09

**Authors:** Cristina Nica, Zhikai Lin, Anton Sculean, Maria B. Asparuhova

**Affiliations:** 1 Laboratory of Oral Cell Biology, Dental Research Center, School of Dental Medicine, University of Bern, Freiburgstrasse 3, 3010 Bern, Switzerland; cristina-gabriela.nica@students.unibe.ch (C.N.); zhikai.lin@zmk.unibe.ch (Z.L.); 2 Department of Periodontology, School of Dental Medicine, University of Bern, Freiburgstrasse 7, 3010 Bern, Switzerland; anton.sculean@zmk.unibe.ch; 3 Department of Periodontology, Shanghai Ninth People’s Hospital, School of Medicine, Shanghai Jiaotong University, Zhizaoju Road 639, Shanghai 200011, China

**Keywords:** biomaterials, xenografts, connective tissue grafts, bone and soft tissue regeneration, growth factors

## Abstract

Xenogeneic acellular collagen matrices represent a safe alternative to autologous soft tissue transplants in periodontology and implant dentistry. Here, we aimed to investigate the adsorption and release of growth factors from four porcine-derived collagen matrices using enzyme-linked immunosorbent assay. Non-crosslinked collagen matrix (NCM), crosslinked collagen matrix (CCM), dried acellular dermal matrix (DADM), and hydrated acellular dermal matrix (HADM) adsorbed each of the following growth factors, TGF-β1, FGF-2, PDGF-BB, GDF-5 and BMP-2, with an efficiency close to 100%. Growth factor release for a 13-day period was in the range of 10–50% of the adsorbed protein, except for the BMP-2 release that was in the range of 5–7%. Generally, protein release occurred in two phases. Phase I was arbitrary defined by the highest release from the matrices, usually within 24 h. Phase II, spanning the period immediately after the peak release until day 13, corresponded to the delayed release of the growth factors from the deeper layers of the matrices. HADM showed significantly (P < 0.001) higher TGF-β1, FGF-2, and PDGF-BB release in phase II, compared to the rest of the matrices. NCM exhibited significantly (P < 0.001) higher FGF-2 release in phase II, compared to CCM and DADM as well as a characteristic second peak in PDGF-BB release towards the middle of the tested period. In contrast to NCM and HADM, CCM and DADM showed a gradual and significantly higher release of GDF-5 in the second phase. Several burst releases of BMP-2 were characteristic for all matrices. The efficient adsorption and sustained protein release in the first 13 days, and the kinetics seen for HADM, with a burst release within hours and high amount of released growth factor within a secondary phase, may be beneficial for the long-term tissue regeneration following reconstructive periodontal surgery.

## 1. Introduction

Although various attempts to augment oral soft tissues using xenogeneic materials have been reported, no ideal substitute material is currently considered to be available and predictable for periodontal and peri-implant plastic surgical reconstructions [1]. The use of xenogeneic materials is of particular advantage in situations where autologous transplants cannot be harvested in sufficient amount or quality, e.g., in thin biotype, shallow palate, or covering of multiple recessions [2,3]. In addition, autogenous soft tissue grafting procedures are usually associated with significant morbidity at the patient donor site [4].

Ideally, a non-autologous graft for soft tissue augmentation should promote hemostasis, be infection-resistant, favor the formation of granulation tissue, and have a low post-operative morbidity and a fast healing time [5]. Acellular collagen matrices of porcine origin offer a safe alternative to autologous soft tissue transplants such as connective tissue grafts and free gingival grafts in a diverse range of indications in periodontology and implant dentistry [2,3]. This includes the treatment of gingival recessions, soft tissue grafting in combination with guided bone regeneration (GBR)/guided tissue regeneration (GTR), gain of attached gingiva, sealing of extraction sockets, and thickening of peri-implant soft tissues. Naturally derived collagen-based materials have attracted a great deal of attention since collagen is a principal component of the connective tissue and may provide structural support. Collagen matrices are capable of facilitating blood clot formation, through hemostatic wound coverage yet remain fully resorbable over time [6,7,8]. Furthermore, crosslinking of the collagen scaffolds is an effective method to improve their mechanical properties and stability [9]. A number of porcine-derived collagen matrices, demonstrating high biocompatibility, sufficient physiochemical stability, and a 3D structure favoring periodontal tissue regeneration, have been developed in the last decade [10,11,12,13]. Preclinical and clinical studies indicate that acellular collagen matrices have been successfully applied in root coverage [14,15,16] as well as in non-root coverage procedures [17,18,19], with low patient morbidity.

Bone and soft tissue regeneration are remarkable regenerative processes initiated by recruitment and differentiation of progenitor cells along with inflammatory cells in order to form granulation tissue, followed by its remodeling into normal bone structure or soft tissue. Both soft tissue wound-healing and bone regeneration are initiated by the formation of a fibrin clot. In the clots, activated platelets release a myriad of growth factors and cytokines/chemokines that stimulate the migration and proliferation of repair cells [20,21]. Among these factors are transforming growth factor-β1 (TGF-β1) and various isoforms of platelet-derived growth factor (PDGF). A number of other growth factors, such as fibroblast growth factor-2 (FGF-2), growth and differentiation factor-5 (GDF-5), and bone morphogenetic protein-2 (BMP-2), are shown to coordinate and control the regenerative process [22].

Collagen matrices represent a promising carrier for growth factors since collagen can bind them [7,23,24,25,26]. For example, resorbable collagen sponges rapidly bind recombinant BMP-2 and BMP-7 and serve as carriers for the growth factors in preclinical and clinical settings [27,28,29]. BMP-2 is a highly osteogenic member of the TGF-β superfamily of proteins, which appears to be a promising alternative to autogenous bone graft for alveolar ridge augmentation [30]. TGF-β1, as a member of the same family, can also bind to collagen and itself stimulate extracellular matrix production and cell proliferation [23,31]. Furthermore, TGF-β-like activity can be passively released from enamel matrix derivative (EMD)-coated collagen products [26].

FGF-2 has exhibited beneficial effects for restoring local blood flow and improving wound-healing in both animal models and clinical studies [32,33]. Furthermore, FGF-2 promotes the migration of fibroblasts and stimulates them to produce collagenase, thus activating the tissue remodeling process [34].

PDGF is a polypeptide growth factor that is secreted by activated platelets early after tissue injury [35]. Its potent effects as a chemoattractant [36] and mitogen [37,38], along with its ability to promote angiogenesis [39], place it as a key regulatory molecule in tissue repair. PDGF has been studied in a variety of preclinical models for safety and tissue regeneration [40,41] as well as in clinical trials in periodontal and orthopedic patients [39,42,43]. PDGF-BB is an isoform of PDGF that is able to activate all three PDGF receptor isoforms (PDGFR-αα, PDGFR-αβ, and PDGFR-ββ) and has been approved for clinical application in periodontal tissue regeneration [38,44].

GDF-5 is another member of the TGF-β superfamily that has been considered as a potential therapeutic agent for periodontal indications [45,46,47]. Osteopromotive procedures combining GDF-5 with β-tricalcium phosphate (β-TCP) have been successfully tested in sinus floor augmentation [48], in peri-implant bone defects in the mandible [49,50], and in advanced intra-bony periodontal defects [51]. It has been demonstrated that recombinant GDF-5 in a β-TCP carrier was more effective in enhancing bone and osteoid formation than recombinant PDGF-BB in the same carrier [52].

Despite the great potential of the listed growth factors for tissue regeneration, the half-life of most of them within the blood is in the range of minutes [53,54], indicating that sustained local delivery of the growth factors is critical in achieving clinical success. To overcome the limitation of the short half-life, biomaterials are often used as carriers of the growth factors. The combination of a biomaterial and a biological agent has been proven as an efficient treatment procedure in periodontal regeneration and plastic surgery [55,56].

Because the adsorption of proteins to the surface of biomaterials is a primary determining factor influencing downstream cellular behavior, the goal of the present study was to investigate the amount of growth factors adsorbed to four commercially available collagen matrices using an enzyme-linked immunosorbent assay (ELISA) for protein quantification. Subsequently, the release kinetics of the growth factors over a 13-day period were quantified.

## 2. Materials and Methods

### 2.1. Porcine-Derived Collagen Matrices and Their Exposure to EMD and Recombinant Growth Factors

The collagen matrices utilized in the current study were: (1) a non-crosslinked collagen matrix, abbreviated NCM (Geistlich Mucograft^®^; Geistlich, Wolhusen, Switzerland); (2) a sugar-crosslinked collagen matrix, abbreviated CCM (Ossix^®^ Volumax; Datum Dental Ltd., Lod, Israel); (3) acellular dermal matrix supplied in a dry form, abbreviated DADM (mucoderm^®^; botiss biomaterials GmbH, Zossen, Germany); and (4) a pre-hydrated acellular dermal matrix consisted of tissue-engineered porcine material, abbreviated HADM (NovoMatrix™ Reconstructive Tissue Matrix; BioHorizons, Birmingham, AL, USA).

Collagen matrices of 10 × 10 mm were incubated for 10 min at room temperature with 1 mL of EMD (Straumann^®^ Emdogain^®^; botiss biomaterials GmbH, Zossen, Germany). Commercial 30 mg/mL EMD was dissolved in 0.1% acetic acid to a stock solution of 10 mg/mL. For coating experiments, the stock EMD was diluted 10-fold in phosphate-buffered saline (PBS), pH 7.2 to a working concentration of 1 mg/mL. Each of the matrices with a size of 10 × 10 mm was also incubated for 10 min at room temperature with 100 ng/mL of each of the following recombinant growth factors: TGF-β1, FGF-2, PDGF-BB, GDF-5, and BMP-2 (all from PeproTech, London, UK). This concentration was chosen based on pilot experiments (data not shown) comparing different concentrations of the growth factors in the range of 25–500 ng/mL. We aimed at obtaining detectable levels of the released growth factors falling into the dynamic range of the ELISA assays. Using the same growth factor concentration for coating of the different matrices allowed various comparisons to be made. As a negative control, membranes were incubated in PBS alone. After the coating, the collagen matrices were rigorously washed with PBS for three cycles of 5 min each.

### 2.2. ELISA Quantification of Protein Adsorption to Collagen Matrices

To determine the quantity of growth factor adsorption to collagen matrices, colorimetric sandwich ELISA Kits from R&D Systems (Bio-Techne AG, Zug, Switzerland) for TGF-β1, FGF-2, PDGF-BB, GDF-5, or BMP-2 were used. Briefly, after a 10-min coating period, the remaining PBS solution containing unattached growth factor as well as the PBS solution from the three washing cycles was collected and quantified by ELISA for the remaining amount of growth factor unadsorbed to the matrix. The ELISAs were performed according to the protocol of the manufacturer. The adsorption was read by using a microplate reader set at a wavelength of 450 nm. Growth factor adsorption by the matrices was quantified by subtracting the unbound growth factor amount from the total amount of protein initially added to the matrices (100 ng) and was expressed in percent. All samples were quantified in triplicate, and three independent experiments were performed.

### 2.3. Analysis of Protein Release Kinetics from Collagen Matrices over Time

In vitro analysis of growth factor release from each of the collagen matrices was performed by incubating the matrices in 3 mL PBS, pH 7.2 containing 0.1% bovine serum albumin (BSA; Sigma, Buchs, Switzerland) and 1% antibiotics/antimycotics (AA; ThermoFisher Scientific, Basel, Switzerland) solution at 37 °C with gentle shaking at 70 rpm for 13 days. Protein quantification by ELISA was performed 0.5, 1, 2, 3, and 6 h, and 1, 3, 5, 7, 9, 11, and 13 days after growth factor loading. At each time point, the 3 mL of supernatant were collected, frozen in low protein-binding tubes (Eppendorf, Basel, Switzerland), and replaced with 3 mL of fresh PBS/BSA/AA solution. The amount of released protein was calculated as percent of the adsorbed protein. Earlier studies identified TGF-β1 in EMD by immunoassays [57] and had shown that TGF-β-like activity can be passively released from EMD-coated collagen products [26], which allowed us to test the release of TGF-β1 from EMD-coated collagen matrices. Three independent experiments with three replicates were performed for each experimental group.

### 2.4. Statistical Analysis

All data are represented as means ± SD. Statistical analysis was completed using GraphPad InStat Software, version 3.05 (GraphPad Software, San Diego, CA, USA). Multiple comparisons were performed using one-way analysis of variance (ANOVA) with Tukey’s post-hoc test. Values of P < 0.05 were considered significant.

## 3. Results

### 3.1. Adsorption Rate and Total Protein Release from Collagen Matrices Over 13-Day Period

**Figure 1:**
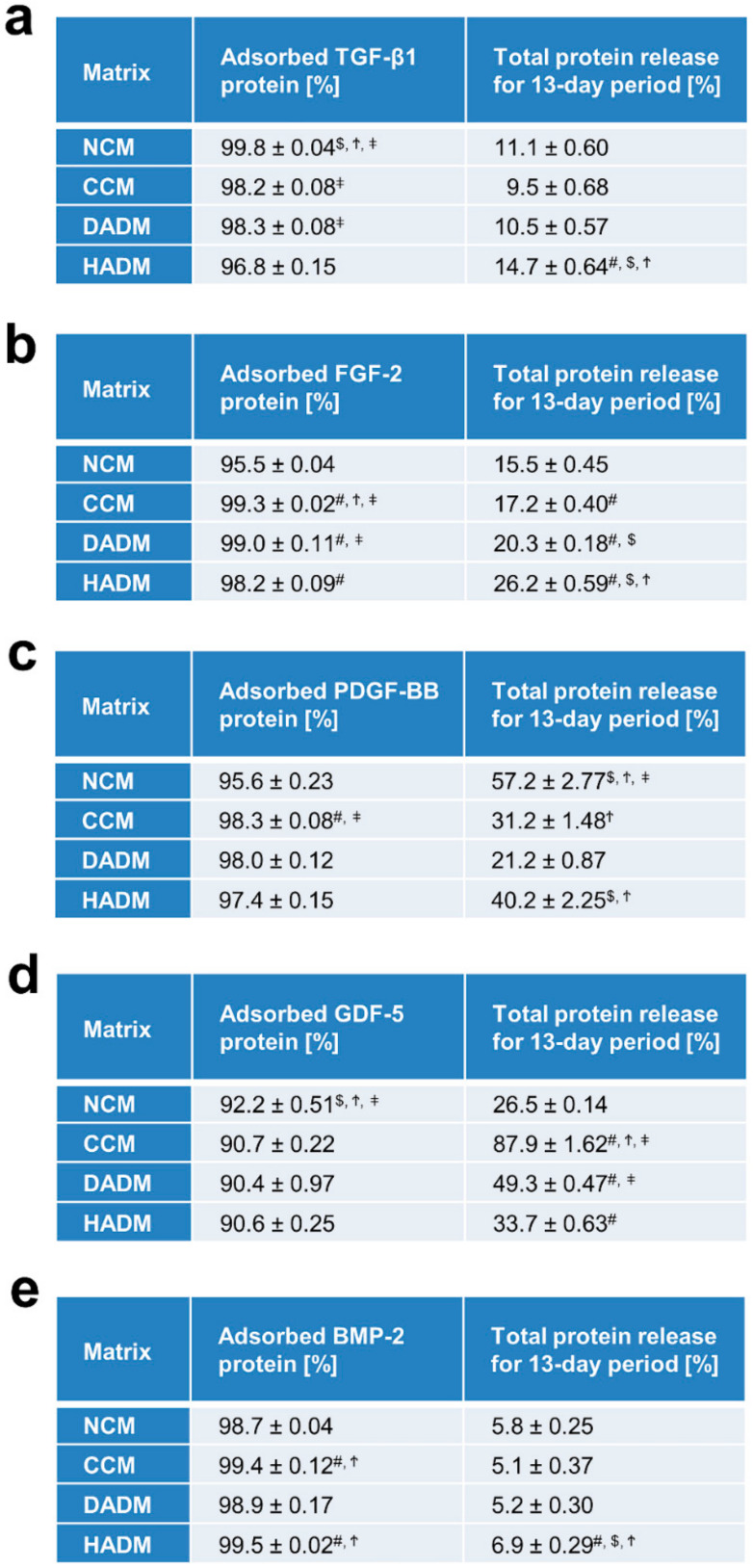
Adsorption rate and total protein release from collagen matrices over a 13-day period. Tables (**a**–**e**) represent quantifications of adsorbed protein (in percent) and total protein release (expressed as percent of adsorbed protein) for a 13-day period from four different collagen matrices. The matrices were abbreviated as follows: NCM (non-crosslinked collagen matrix; Geistlich Mucograft^®^), CCM (crosslinked collagen matrix, Ossix^®^ Volumax), DADM (dried acellular dermal matrix, mucoderm^®^), and HADM (hydrated acellular dermal matrix; NovoMatrix™ Reconstructive Tissue Matrix). Matrices were incubated for 10 min at room temperature in PBS containing 100 ng/mL of each of the following recombinant growth factors: TGF-β1 (**a**), FGF-2 (**b**), PDGF-BB (**c**), GDF-5 (**d**), and BMP-2 (**e**). Protein quantifications were performed by using colorimetric ELISA assays. Means ± SD from three independent experiments and significant differences (P < 0.05) between the experimental groups are shown. Significance was indicated with the following symbols: # denotes significantly higher than NCM, $ denotes significantly higher than CCM, Ϯ denotes significantly higher than DADM, and ǂ denotes significantly higher than HADM.

**Figure 2:**
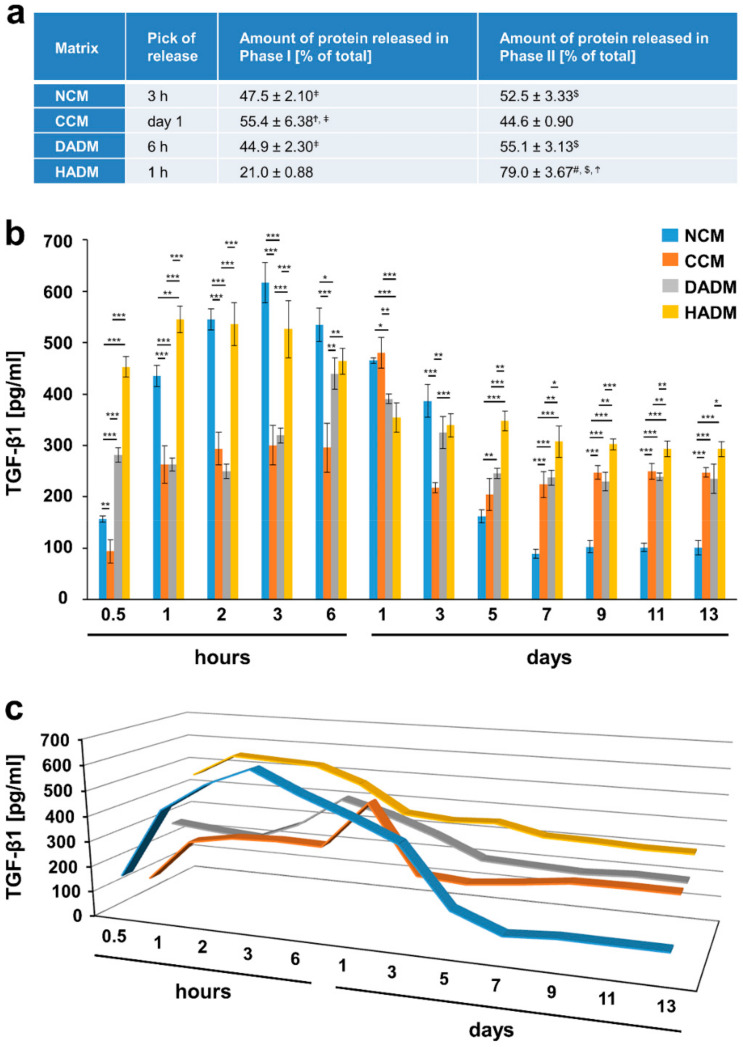
In vitro release of TGF-β1 from recombinant TGF-β1-coated collagen matrices over time. (**a**) The table represents: (1) the time point at which the highest TGF-β1 release from each of the four collagen matrices, coated with recombinant TGF-β1, was observed (peak of the release); (2) ELISA quantifications of the TGF-β1 released in phase I and expressed as percent of the total protein release for the entire test period of 13 days (taken as 100%); (3) ELISA quantifications of the TGF-β1 released in phase II and expressed as in (2). The peak of the protein release marks the end of phase I whereas phase II spans the time period immediately after the peak of the release until day 13. Means ± SD from three independent experiments and significant differences (P < 0.05) between the experimental groups are shown. Significance was indicated with the following symbols: # denotes significantly higher than NCM, $ denotes significantly higher than CCM, Ϯ denotes significantly higher than DADM, and ǂ denotes significantly higher than HADM. (**b**) ELISAs were used to quantify the amounts of TGF-β1 in conditioned PBS solution collected from the matrices at the indicated time intervals over a 13-day period. Data represent means ± SD from three independent experiments. Significant differences between experimental groups, *** P < 0.001, ** P < 0.01, * P < 0.05. (**c**) Three-dimensional graph of the data present in (**b**) for better visualization of the release kinetics.

**Figure 3:**
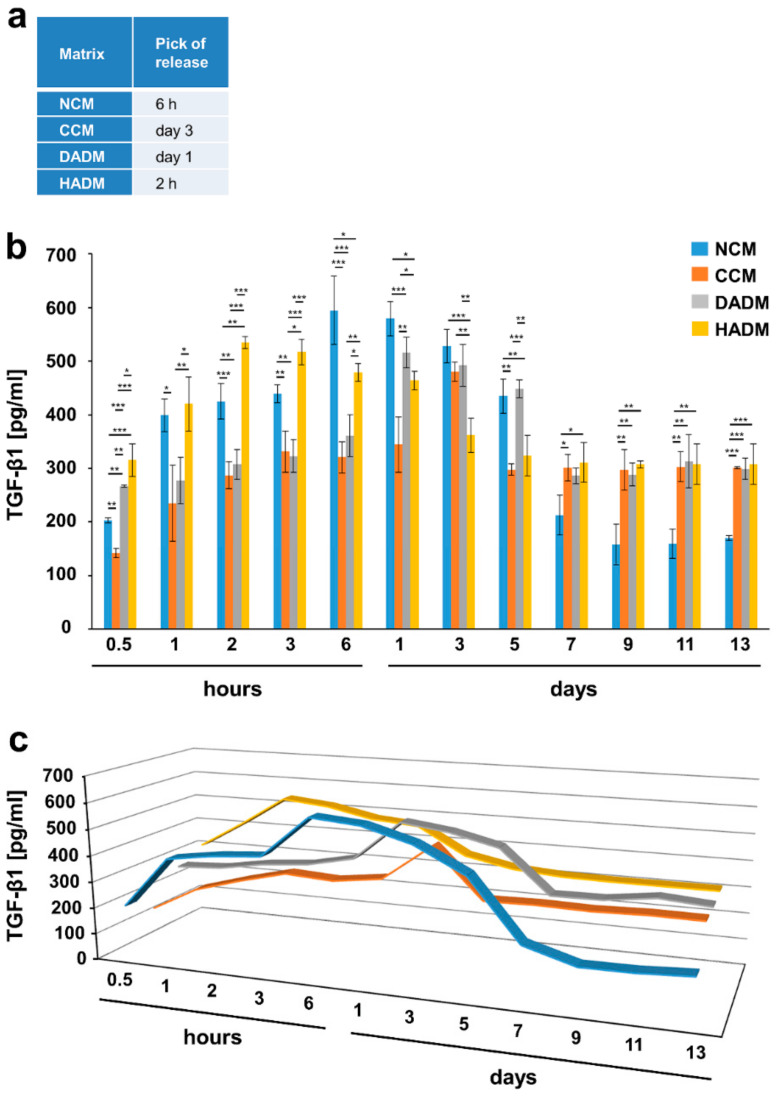
In vitro release of TGF-β1 from EMD-coated collagen matrices over time. (**a**) The table represents the time point at which the highest TGF-β1 protein release from each of the four collagen matrices, coated with EMD, was observed (peak of the release). (**b**) ELISAs were used to quantify the amounts of TGF-β1 in conditioned PBS solution collected from the matrices at the indicated time intervals over a 13-day period. Data represent means ± SD from three independent experiments. Significant differences between experimental groups, *** P < 0.001, ** P < 0.01, * P < 0.05. (**c**) Three-dimensional graph of the data present in (**b**) for better visualization of the release kinetics.

Analyses of the adsorption rate of recombinant growth factors to collagen matrices have indicated that each of the four investigated collagen matrices adsorbed each of the five growth factors with a great efficiency, close to 100%, after a 10-min coating period and extensive washing with PBS (Figure 1). An exception was seen for the adsorption of recombinant GDF-5, with an efficiency in the range of 90%–92% (Figure 1d). Across all matrices and growth factors, an average of 3.1% of the protein remained unabsorbed or was washed out during the rinsing process. The adsorbed TGF-β1 and GDF-5 on NCM was slightly but significantly higher compared to the adsorption of the two growth factors on CCM, DADM, or HADM (Figure 1a,d). The protein adsorption on the sugar-crosslinked CCM was significantly higher for: (1) TGF-β1 compared to its adsorption on HADM (Figure 1a); (2) FGF-2 compared to its adsorption on NCM, DADM, and HADM (Figure 1b); (3) PDGF-BB compared to its adsorption on NCM and HADM (Figure 1c); and (4) BMP-2 compared to its adsorption on NCM and DADM (Figure 1e). The observed efficient adsorption on CCM does not suggest a negative influence of the collagen crosslinking of the matrices on the adsorption rate of the tested recombinant growth factors. Although the differences were minor, DADM adsorbed significantly higher quantities of TGF-β1 compared to HADM (Figure 1a), as well as higher FGF-2 quantities compared to NCM and HADM (Figure 1b). Finally, HADM adsorbed significantly higher quantities of FGF-2 compared to NCM (Figure 1b) as well as significantly higher amounts of BMP-2 compared to both NCM and DADM (Figure 1e).

The total growth factor release from the different collagen matrices, expressed as percent of the adsorbed protein, for the entire test period of 13 days was ranging from 5–7% for BMP-2 from each of the matrices (Figure 1e), up to 88% for GDF-5 from CCM (Figure 1e). A release below 10% after 13 days, we arbitrarily classified as a low release. A release between 10% and 50% was classified as a moderate and above 50% as a high release. Thus, the release of TGF-β1 as well as of FGF-2 was moderate for all collagen matrices coated with the respective factors (Figure 1a,b). In particular, the total growth factor release from TGF-β1-coated matrices for the entire test period was in the range of 10–15%, with significantly more pronounced release from HADM compared to the other three matrix products (Figure 1a). The total FGF-2 protein release was in the range of 16–26% and was increasing with statistically significant differences in the following sequence: NCM–CCM–DADM–HADM (Figure 1b). In contrast, PDGF-BB and GDF-5 showed greater variability in their release for the 13-day period across the four matrices (Figure 1c,d). A moderate PDGF-BB release below 50% for the entire 13-day period was detected for DADM (21.2% ± 0.87%), CCM (31.2% ± 1.48%) and HADM (40.2% ± 2.25%), whereas a significantly higher release was observed for NCM (57.2% ± 2.77%) (Figure 1c). On contrary, the GDF-5 release occurred at a moderate rate for NCM (26.5% ± 0.14%), HADM (33.7% ± 0.63%) and DADM (49.3% ± 0.47%), whereas CCM showed a significantly higher release close to 90% (Figure 1d).

The data obtained across the different matrices and growth factors tested in the present study do not suggest a correlation between the crosslinking of the collagen in the matrices and the speed of release of adsorbed growth factors by the matrices. Furthermore, with some exceptions, the total protein release for the tested 13-day period was relatively low compared to the adsorbed protein amounts, especially for BMP-2, suggesting that release of the growth factors from the investigated matrix products continues over a period longer than 13 days.

### 3.2. Detailed Analysis of the Release of TGF-β1 from Recombinant TGF-β1- or EMD-Coated Collagen Matrices over Time

**Figure 4:**
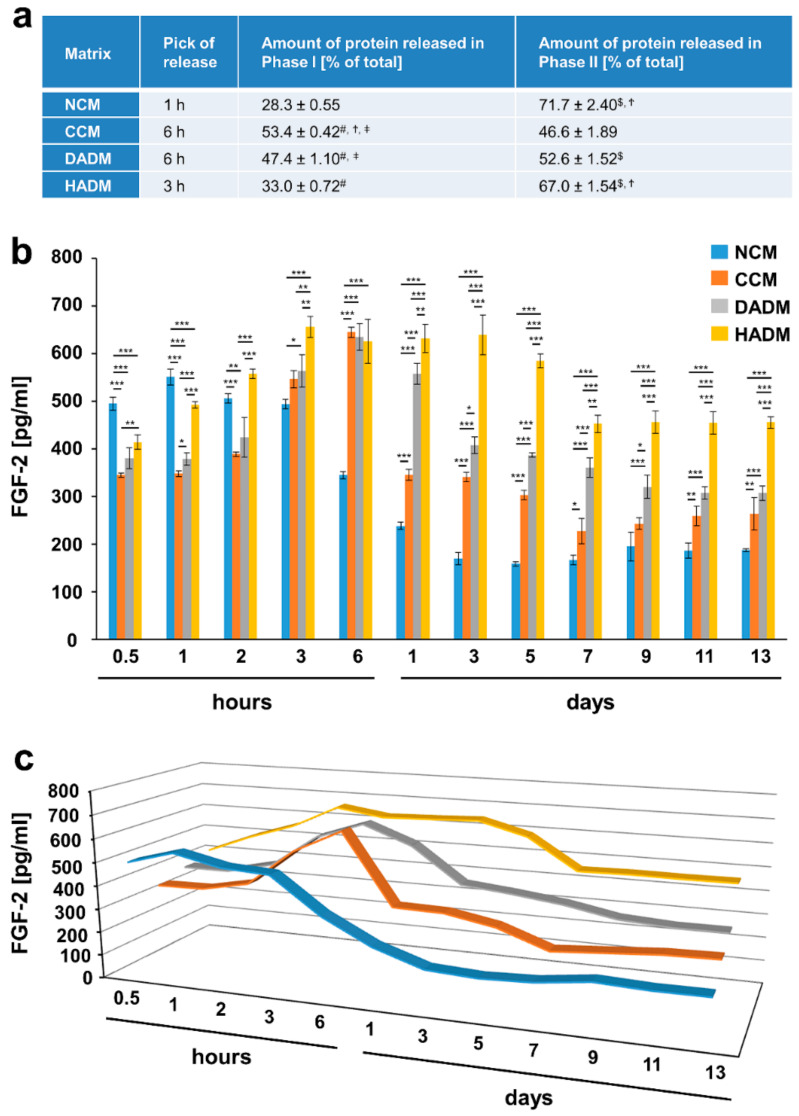
In vitro release of FGF-2 from recombinant FGF-2-coated collagen matrices over time. (**a**) The table represents: 1) the time point at which the highest FGF-2 release from each of the four collagen matrices, coated with recombinant FGF-2, was observed (peak of the release); 2) ELISA quantifications of the FGF-2 released in phase I and expressed as percent of the total protein release for the entire test period of 13 days (taken as 100%); 3) ELISA quantifications of the FGF-2 released in phase II and expressed as in 2). The peak of the protein release marks the end of phase I whereas phase II spans the time period immediately after the peak of the release until day 13. Means ± SD from three independent experiments and significant differences (P < 0.05) between the experimental groups are shown. Significance was indicated with the following symbols: # denotes significantly higher than NCM, $ denotes significantly higher than CCM, Ϯ denotes significantly higher than DADM, and ǂ denotes significantly higher than HADM. (**b**) ELISAs were used to quantify the amounts of FGF-2 in conditioned PBS solution collected from the matrices at the indicated time intervals over a 13-day period. Data represent means ± SD from three independent experiments. Significant differences between experimental groups, *** P < 0.001, ** P < 0.01, * P < 0.05. (**c**) Three-dimensional graph of the data present in (**b**) for better visualization of the release kinetics.

**Figure 5:**
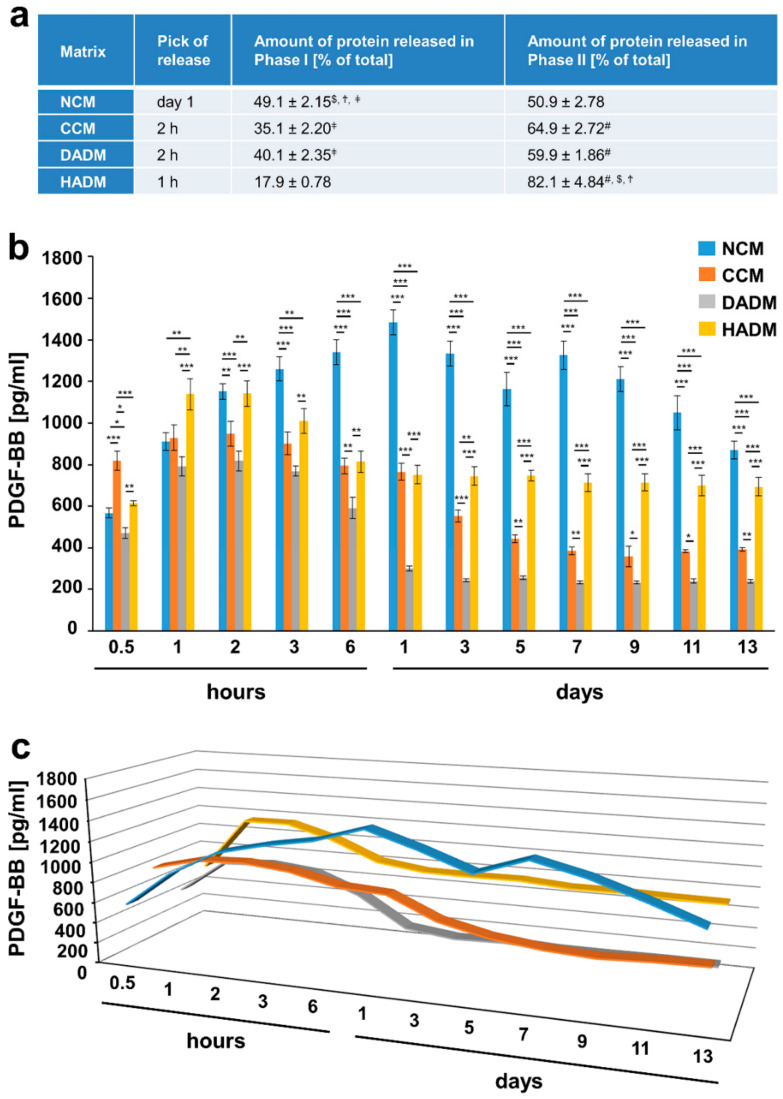
In vitro release of PDGF-BB from recombinant PDGF-BB-coated collagen matrices over time. (**a**) The table represents: (1) the time point at which the highest PDGF-BB protein release from each of the four collagen matrices, coated with recombinant PDGF-BB, was observed (peak of the release); 2) ELISA quantifications of the PDGF-BB released in phase I and expressed as percent of the total protein release for the entire test period of 13 days (taken as 100%); (3) ELISA quantifications of the PDGF-BB released in phase II and expressed as in (2). The peak of the protein release marks the end of phase I whereas phase II spans the time period immediately after the peak in the release until day 13. Means ± SD from three independent experiments and significant differences (P < 0.05) between the experimental groups are shown. Significance was indicated with the following symbols: # denotes significantly higher than NCM, $ denotes significantly higher than CCM, Ϯ denotes significantly higher than DADM, and ǂ denotes significantly higher than HADM. (**b**) ELISAs were used to quantify the amounts of PDGF-BB in conditioned PBS solution collected from the matrices at the indicated time intervals over a 13-day period. Data represent means ± SD from three independent experiments. Significant differences between experimental groups, *** P < 0.001, ** P < 0.01, * P < 0.05. (**c**) Three-dimensional graph of the data present in (**b**) for better visualization of the release kinetics.

**Figure 6:**
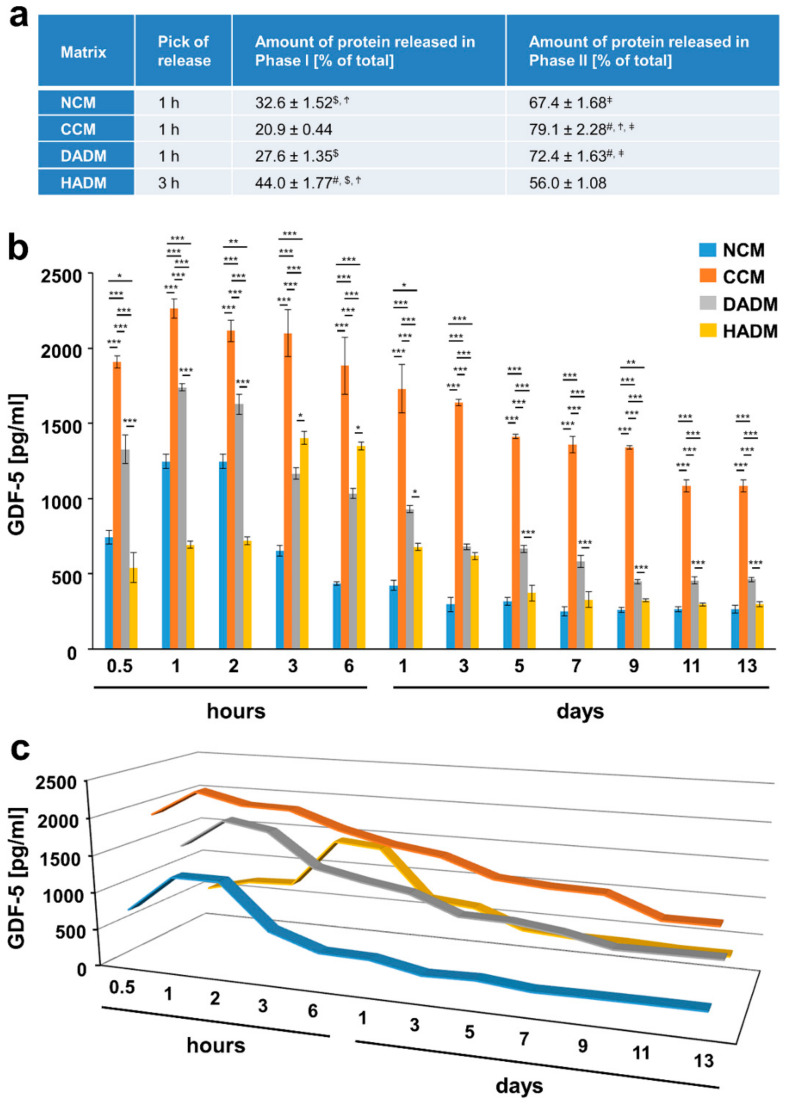
In vitro release of GDF-5 from recombinant GDF-5-coated collagen matrices over time. (**a**) The table represents: (1) the time point at which the highest GDF-5 protein release from each of the four collagen matrices, coated with recombinant GDF-5, was observed (peak in the release); (2) ELISA quantifications of the GDF-5 released in phase I and expressed as percent of the total protein release for the entire test period of 13 days (taken as 100%); (3) ELISA quantifications of the GDF-5 released in phase II and expressed as in (2). The peak of the protein release marks the end of phase I whereas phase II spans the time period immediately after the peak in the release until day 13. Means ± SD from three independent experiments and significant differences (P < 0.05) between the experimental groups are shown. Significance was indicated with the following symbols: # denotes significantly higher than NCM, $ denotes significantly higher than CCM, Ϯ denotes significantly higher than DADM, and ǂ denotes significantly higher than HADM. (**b**) ELISAs were used to quantify the amounts of GDF-5 in conditioned PBS solution collected from the matrices at the indicated time intervals over a 13-day period. Data represent means ± SD from three independent experiments. Significant differences between experimental groups, *** P < 0.001, ** P < 0.01, * P < 0.05. (**c**) Three-dimensional graph of the data present in (**b**) for better visualization of the release kinetics.

**Figure 7:**
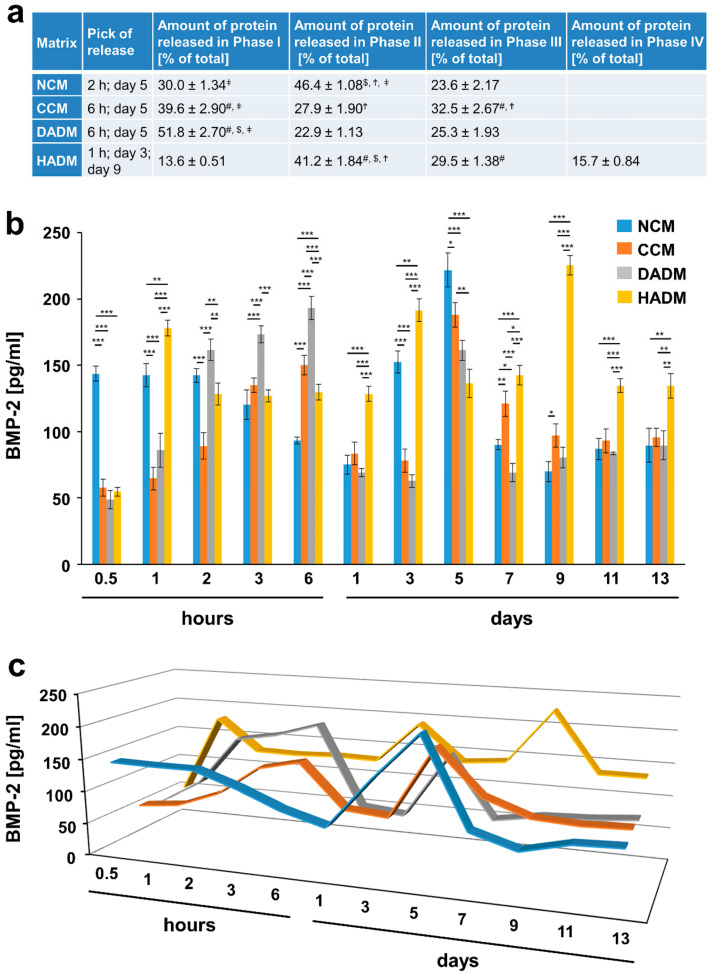
In vitro release of BMP-2 from recombinant BMP-2-coated collagen matrices over time. (**a**) The table represents: (1) the time point at which the highest BMP-2 protein release from each of the four collagen matrices, coated with recombinant BMP-2 was observed (peak in the release); (2) ELISA quantifications of the BMP-2 released in phase I and expressed as percent of the total protein release for the entire test period of 13 days (taken as 100%); (3) ELISA quantifications of the BMP-2 released in phase II and expressed as in (2). The peak in the protein release marks the end of phase I whereas phase II spans the time period immediately after the peak of the release until day 13. Means ± SD from three independent experiments and significant differences (P < 0.05) between the experimental groups are shown. Significance was indicated with the following symbols: # denotes significantly higher than NCM, $ denotes significantly higher than CCM, Ϯ denotes significantly higher than DADM, and ǂ denotes significantly higher than HADM. (**b**) ELISAs were used to quantify the amounts of BMP-2 in conditioned PBS solution collected from the matrices at the indicated time intervals over a 13-day period. Data represent means ± SD from three independent experiments. Significant differences between experimental groups, *** P < 0.001, ** P < 0.01, * P < 0.05. (**c**) Three-dimensional graph of the data present in (**b**) for better visualization of the release kinetics.

Except for BMP-2, the release of the adsorbed growth factors from the collagen matrices, after a 10-min coating period and extensive washing, occurred in two phases. The peak of the protein release marked the end of phase I and was detected at an early time point, usually within 24 h. The second phase, spanning the time period immediately after the peak of the release until day 13, was characterized by a sustained slow release of the growth factors (Figure 2, Figure 3, Figure 4, Figure 5, Figure 6 and Figure 7).

In particular, the highest release of TGF-β1 from TGF-β1-coated collagen matrices was observed within 1 h for the HADM, 3 h for the NCM, 6 h for the DADM, and within 1 day for the crosslinked CCM (Figure 2). The highest release of the same growth factor from EMD-coated matrices was shifted towards longer time periods falling again within the first 24 h for all the matrices except for the CCM, where the peak of the release was reached only on day 3 (Figure 3). The peak of the TGF-β1 release from TGF-β1-coated matrices was followed by a sustained slow decrease of the released amount for up to 5 days in the case of the non-crosslinked NCM, DADM and HADM (Figure 2). In contrast, CCM was characterized by a sharp drop in the release of the adsorbed TGF-β1 protein immediately after the peak. The same release kinetic was typical for the release of TGF-β1 from EMD-coated CCM immediately after the peak on day 3 (Figure 3). The peak of the TGF-β1 release from EMD-coated matrices was followed by a sustained slow decrease in the released amount up to 5 days in the case of HADM and up to 7 days in the case of NCM and DADM. The amount of TGF-β1 protein released from TGF-β1-coated NCM, CCM, and DADM during phase II compared to phase I was nearly equal and amounted to approximately 50% of the total released protein (taken as 100%) (Figure 2a). Interestingly, HADM coated with recombinant TGF-β1 was the only matrix that showed significantly (P < 0.001) higher TGF-β1 release (up to 80% of the totally released protein) in the second phase following an initial rapid release within hours, compared to the other three matrices. The latter type of release kinetics with a burst release within 1 h and high amount of released protein within a prolonged secondary phase might be advantageous for the long-term tissue regeneration following reconstructive periodontal surgeries.

### 3.3. Detailed Analysis of the Release of FGF-2 from Recombinant FGF-2-Coated Collagen Matrices over Time

Similarly to the release kinetics of TGF-β1, the release of FGF-2 growth factor burst at early time points, within hours, i.e., at 1 h for NCM, 3 h for HADM, and 6 h for CCM and DADM (Figure 4). The burst release was followed by a quick drop in the released FGF-2 amounts from CCM (~50% decrease at day 1) versus a slightly delayed drop in the release from DADM at day 5, and a sustained slow decrease from NCM and HADM during the second release phase. Whereas the amount of FGF-2 protein released from FGF-2-coated CCM and DADM in phase I and phase II was nearly equal and amounted to approximately 50% of the total protein, a significantly (P < 0.001) higher FGF-2 release amounting to approximately 70% of the total protein was observed for NCM and HADM during the second phase (Figure 4a). The sustained high release (~500 pg/mL) of FGF-2 from NCM continued for 3 h, followed by a slow but substantial decrease in the released amounts by 3-fold. This was likely due to the faster degradation rate of NCM compared to the other matrices. In contrast, high amounts of FGF-2 released from HADM in the range of 630 pg/mL were maintained between the peak of the release at 3 h until day 7, when a small decrease in the released quantities of only 1.3-fold was observed and the respective amounts were maintained until day 13 (Figure 4b,c).

Thus, our data confirm HADM as the matrix with the most favorable release kinetic of FGF-2 along with TGF-β1 growth factor.

### 3.4. Detailed Analysis of the Release of PDGF-BB from Recombinant PDGF-BB-Coated Collagen Matrices over Time

The highest release of PDGF-BB from PDGF-BB-coated collagen matrices was observed within 1 h for the HADM, 2 h for the DADM and CCM, and within 1 day for the NCM (Figure 5). Similarly to the release kinetics of TGF-β1 and FGF-2, the highest amount of PDGF-BB, equal to 82.1% of the total protein released within the 13-day period, was observed in the second release phase for HADM, followed by CCM (64.9% ± 2.72%) and DADM (59.9% ± 1.86%) (Figure 5a). The amount of PDGF-BB protein released from PDGF-BB-coated matrices in phase I and phase II was nearly equal and amounted to approximately 50% of the total protein released from NCM. Compared to the rest of the matrices, NCM released the highest amount of adsorbed PDGF-BB (1484 pg/mL) via a gradual increase within 24 h, followed by an equivalent gradual decrease and a second release peak at day 7 likely corresponding to the delayed release of the growth factor from the deeper layers of the matrices (Figure 5b,c). HADM was characterized by a sustained and very slow release of PDGF-BB with no decrease in the released quantities of the growth factor between day 1 and day 13. In contrast, DADM and CCM showed a gradual decrease up to day 1 and 5, respectively, when a sharper drop in the released PDGF-BB quantities were observed.

The obtained results delineate NCM and HADM as good carriers for recombinant PDGF-BB due to: (1) a gradual increase in the released amounts within 24 h and a second burst release at day 7 in the case of NCM; and (2) an early burst in the release within 1 h and a sustained slow release thereafter in the case of HADM.

### 3.5. Detailed Analysis of the Release of GDF-5 from Recombinant GDF-5-Coated Collagen Matrices over Time

Among the investigated growth factors, GDF-5 showed the earliest peak of release from all four collagen matrices, i.e., at 1 h for NCM, CCM, and DADM, and 3 h for HADM (Figure 6). Thus, it is also the growth factor showing a prolonged secondary phase with a significantly higher release from all collagen matrices, equaling to more than 70% of the total released protein for CCM and DADM (Figure 6a). Both CCM and DADM showed a gradual release of GDF-5 compared to the observed drop in the released protein soon after the burst release, which was typical for NCM and HADM (Figure 6b,c). In addition, CCM was characterized by the release of extremely high quantities of GDF-5 (2′265 pg/mL at 1 h), which in combination with its gradual release contributed to the fact that nearly all adsorbed protein was released in the surrounding media for the tested 13-day period (Figure 1d).

### 3.6. Detailed Analysis of the Release of BMP-2 from Recombinant BMP-2-coated Collagen Matrices over Time

The highly osteogenic growth factor BMP-2 exhibited a continuous slow increase in its release from NCM, CCM, and DADM between 2 and 6 h, followed by a sharp decrease, and a second burst release at day 5 (Figure 7). Thus, between 68% and 76% of the BMP-2 was released in the surrounding medium from NCM, CCM, and DADM in the first 5 days. Interestingly, compared to the other matrices, HADM showed earlier release peaks at 1 h and 3 days and in addition a third pick at day 9, with 84.3% of the growth factor being delivered in the medium within 9 days.

In conclusion, the small amount of total BMP-2 released during the entire 13-day period (Figure 1e) in combination with several time points at which a burst release was observed might be advantageous for the slow process of hard tissue regeneration following implant placement or periodontal reconstruction.

## 4. Discussion

A commonly used approach for soft tissue repair in the oral cavity involves the use of an autologous graft taken from a different part of the cavity, most often from the palate [58]. However, in large defects the amount of harvested tissue is limited by the donor site while the harvesting procedure itself increases patient morbidity, an aspect which is especially relevant in more extensive reconstructions [4]. Xenogeneic collagen matrices are recognized as an optimal alternative to autologous grafts in the field of periodontal soft tissue regeneration [2,3]. As a biomaterial, collagen has a number of beneficial properties, including biocompatibility and biodegradability [6,7,8,9,59]. The collagen matrices are able to attract cells of different phenotypes, which sequentially express and secrete factors for soft and hard tissue regeneration including tissue remodeling and vascularization. Among these factors, TGF-β1, FGF-2, PDGF-BB, and BMP-2 play a pivotal role in tissue repair and remodeling. That way, growth factors are either made de novo by cells attracted in the matrix compartment and then adsorbed on the matrices or they are directly adsorbed on the matrices from the surrounding fluids and tissues. In both cases, the important question concerning the physicochemical properties of the matrices is the adsorption rate and the release kinetics of the growth factors from the matrices. Thus, the present study aimed to shed light on the amount of growth factors adsorbed to four different collagen matrices and the release kinetics of the growth factors over a 13-day period. The adsorption of EMD or recombinant growth factors to xenogeneic collagen matrices is considered to exert beneficial effects on the periodontal regeneration. The reason for this is the potential additive effects of the combination approach compared to the effects of the single components [60].

In the current study, we made the choice to investigate four commercially available xenogeneic matrices. NCM was included in the investigation because of its widespread use in regenerative dentistry. It is a non-crosslinked, resorbable, porcine bilayered matrix composed of collagen types I and III [10,61]. While NCM represents native collagen, CCM is consisted of sugar-crosslinked collagen type I. DADM and HADM are provided in dried and pre-hydrated form, respectively, but both are derived from porcine dermis. DADM is obtained after a multi-step process aimed at removing antigenic components [11,62]. It has been suggested as a carrier for EMD in the treatment of gingival recessions [62]. The study suggests a synergistic effect of both on soft tissue regeneration and the formation of root cementum in recession-type defects. HADM is a reconstructive tissue matrix of new generation. Proprietary tissue processing retains components such as fibrillar collagens (types I, II, III, V, XI, XXIV and XXVII) and collagen type VI, fibronectin, elastin, hyaluronan, and proteoglycans, all of which appear critical to maintaining biochemical and biomechanical tissue integrity. A recent in vivo study, performed in beagle dogs, has compared NCM and HADM for their ability to treat gingival recession defects [63]. Importantly, both porcine-derived collagen matrices revealed similar histological outcomes with successful integration and absence of adverse events. However, the HADM provided superior outcomes regarding root coverage and tissue thickness.

To the best of our knowledge, the present study represents the first comparative in vitro study of the matrices described above. Each of the investigated matrices is designed for a range of particular clinical settings. A common indication is the GTR for recession coverage. However, the clinically relevant differences in the intended usage of the four matrices are not supposed to affect the capacity of the collagen to adsorb EMD or recombinant growth factors. In support of this concept, no discerning differences in terms of absorbency were observed among the four collagen matrices. Indeed, our data demonstrate an efficient adsorption, close to 100%, for all five recombinant growth factors on each of the investigated collagen matrices after a clinically relevant, short incubation period of 10 min.

With few exceptions, growth factor release seems to occur in two phases. In the first phase, the highest release from the matrices was observed at an early time point, usually within 24 h. The second phase, spanning the time period immediately after the peak in the release until day 13, seems to correspond to the delayed release of the growth factors from the deeper layers of the matrices. All investigated matrices were able to maintain and gradually release adsorbed growth factors for a 13-day period. The growth factors used in the current study have a potent individual and synergistic effects on the different processes involved in periodontal tissue regeneration and alveolar bone repair in the oral cavity [64]. However, these biomolecules have short half-lives and narrow therapeutic windows due to degradation by enzymes, leading to insufficient soft tissue remodeling and/or bone formation [53,54]. On the other hand, the local delivery of excess amounts of these biomolecules is not desirable too, as they may induce malignancy [65]. Thus, the sustained release of growth factors from the investigated matrices over time appears beneficial and justifies their short-term coating with EMD or recombinant growth factors approved for clinical use. In particular, the total release of TGF-β1, FGF-2, PDGF-BB, and GDF-5, expressed as a percent of the adsorbed protein for the entire test period of 13 days, was moderate for nearly all investigated collagen matrices. An exception was seen for the highly osteogenic BMP-2. It showed a very low release, in the range of 5%–7% of the adsorbed protein, for the entire test period, as well as several characteristic burst releases from all investigated collagen matrices. Considering that the healing of bone defects during periodontal regeneration encompasses between 6 and 12 weeks on average [66], the low release of the osteogenic BMP-2 in the first 13 days after growth factor loading is to be considered beneficial for the healing process. BMP-2 as well as FGF-2 are involved in the recruitment and formation of osteoclasts, directly or indirectly via increased angiogenesis and osteogenesis [67,68]. Furthermore, FGF-2 stimulates the migration and proliferation of different cells, including endothelial cells, fibroblasts and osteoblasts, during bone healing [22]. In addition, it plays a critical role in angiogenesis and mesenchymal cell growth [22]. It is therefore likely that FGF-2 and BMP-2 proteins, together with the collagenous support structure of the matrices, are important factors in the migration, proliferation, and differentiation of the cells populating the matrices [69].

Beside growth factors, synthetic peptides, inspired by intrinsically disordered proteins (IDPs) and based on proline-rich domains of enamel matrix proteins, have been shown to stimulate osteoblast differentiation in pre-osteoblastic MC3T3-E1 cells as well as in human mesenchymal stem cells [70,71,72]. These peptides were successfully and safely loaded on bone graft materials and showed enhanced in vitro bone cell responses [73,74]. In vivo, proline-rich peptides mimicked the effects of EMD, accelerating the wound-healing on rat oral mucosa. Furthermore, IDP-coated titanium implants demonstrated enhanced osseointegration by reducing bone resorption [75]. Future research should aim to evaluate the effects of IDP-coated collagen matrices on periodontal regeneration in vitro and in vivo.

A number of the literature reports announced the requirement for surface modifications to biomaterials for the controlled release of growth factors over time, e.g., maintained bioactivity and sustained release of growth factors was achieved using a heparin-mediated delivery system [76,77,78,79]. Nillesen et al. chemically conjugated heparin to porous collagen scaffolds and showed that a homogenous distribution of FGF-2 and vascular endothelial growth factor could be achieved on the heparin-conjugated scaffolds [78]. Furthermore, the interaction with heparin appears to protect the growth factors from denaturation and proteolytic degradation [80]. However, the high adsorption rate and sustained release of the growth factors observed in the present study suggest that surface modification of the investigated collagen matrices is most likely not needed.

The data described in the current study allow further conclusions to be drawn. The matrix of new generation, HADM, has consistently shown a very early burst release within hours, followed by a prolonged secondary phase, characterized by the release of high quantities of TGF-β1, FGF-2 and PDGF-BB, amounting to 70–80% of the total protein release for the entire test period. Considering that TGF-β1, FGF-2 and PDGF-BB are growth factors needed at rather early stages of the wound healing process serving pro-migratory and pro-proliferative functions [81], the release kinetics of HADM are to be considered beneficial for the regenerative process. As a natural collagen matrix, NCM is the only other matrix that behaves similarly to the tissue engineered HADM, in particular for the release of FGF-2, in the frame of a prolonged second release phase. Furthermore, sustained high quantities of PDGF-BB were released from NCM with a characteristic 2-peak release kinetic. CCM as a representative of the crosslinked collagen matrices released gradually extremely high quantities of GDF-5, resulting in the release of nearly the entire amount of adsorbed protein within 13 days. This is likely due to a significantly different degree of GDF-5 penetration and binding strength to CCM compared to the rest of the matrices, resulting in the release of the growth factor predominantly from the superficial layers of the matrix. A kinetic where the entire adsorbed amount of GDF-5 is released in the surrounding media within a relatively short time suggests that a combination of the CCM matrix with another biomaterial, such as a bone substitute, might be advantageous. In this case, the additional biomaterial may adsorb the released growth factor for prolonging its presence and activity at the defect site. Finally, DADM is the matrix showing consistently equal release in both phases except for GDF-5, where the release in the second phase was gradual and equal to 72% of the totally released protein over 13 days. It might be hypothesized that the differences observed in the release rate of the same growth factor from the four collagen matrices might be related to the different microstructures of the matrices. Each of the matrices is characterized by a unique layering and porous structure that requires detailed investigation in a comparative manner.

Since their introduction in the 1990s, numerous studies have investigated the use of acellular dermal matrices similar to the ones used in the current study (DADM and HADM) in periodontal plastic surgery procedures. An increase in root coverage and tissue thickness, as well as augmentation of the keratinized tissue, have been reported after treatment with ADMs [62,82,83]. Non-crosslinked bovine type I collagen loaded with PDGF-BB or GDF-5 has been shown to increase the proliferative and differentiation properties of MC3T3-E1 preosteoblasts in vitro as well as to significantly accelerate bone regeneration in rat mandibular defects in vivo [84]. In the context of these literature reports, the present pilot study is to be considered a pre-requisite for further in vitro and in vivo studies. Further research is needed to assess the impact of the investigated collagen matrices loaded with various growth factors on the behavior of specific cell types involved in soft and hard tissue regeneration in vitro as well as on tissue healing in vivo. However, the present study shows efficient adsorption and release of growth factors from all four collagen matrices, suggesting that functionalization of collagen-based matrices with bioactive substances, growth and differentiation factors might be possible. Furthermore, our findings contribute to the biomaterial research by providing an opportunity to intentionally tailor the composition of xenogeneic matrices to enhance the regenerative processes for specific clinical indications.
